# Promoting a functional and comparative understanding of the conifer genome- implementing applied aspects for more productive and adapted forests (ProCoGen)

**DOI:** 10.1186/1753-6561-5-S7-P158

**Published:** 2011-09-13

**Authors:** Carmen Díaz-Sala, Teresa Cervera

**Affiliations:** 1 Dpto. Biología Vegetal, Universidad de Alcalá, 28871 Alcalá de Henares, Madrid, Spain; 2 Dpto. Ecología y Genetíca Forestal, CIFOR-INIA, 28040 Madrid, Spain

## 

In the midst of a climatic change scenario, the genetics of adaptive response in conifers becomes essential to ensure a sustainable management of genetic resources and an effective breeding. Conifers are the target of major tree breeding efforts worldwide. Advances in molecular technologies, such as next-generation DNA sequencing technologies, could have an enormous impact on the rate of progress and achievements made by tree breeding programmes. These new technologies might be used not only to improve our understanding of fundamental conifer biology, but also to address practical problems for the forest industry as well as problems related to the adaptation and management of conifer forests. In this context, the FP7-KBBE-2011-5 project “Promoting a functional and comparative understanding of the conifer genome- implementing applied aspects for more productive and adapted forests” (ProCoGen), granted in 2011 by the European Commission, will address genome sequencing of two keystone European conifer species. Genome re-sequencing approaches will be used to obtain two reference pine genomes. Comparative genomics and genetic diversity will be closely integrated and linked to targeted functional genomics investigations to identify genes and gene networks that efficiently help to develop or enhance applications related to forest productivity, forest stewardship in response to environmental change or conservation efforts. The development of high-throughput genotyping tools will produce an array of pre-breeding tools to be implemented in forest tree breeding programmes. ProCoGen will also develop comparative studies based on orthologous sequences, genes and markers, which will allow guiding re-sequencing initiatives and exploiting the research accumulated on each of the species under consideration to accelerate the use of genomic tools in diverse species. ProCoGen will integrate fragmented activities developed by European research groups involved in several ongoing international conifer genome initiatives and contribute to strengthening international collaboration with North American initiatives (US and Canada). Partners involved in this project are:

Carmen Díaz-Sala (financial and administrative coordinator, Universidad de Alcalá, UAH, Spain)

María-Teresa Cervera (scientific coordinator, Instituto Nacional de Investigación y Tecnología Agraria y Alimentaria, INIA-CIFOR, also including Toni Gabaldón from Centro de Regulación Genómica, CRG; Álvaro Soto from Universidad Politécnica de Madrid, UPM, and Isabel Arrillaga from Universidad de Valencia, UV, Spain)

Francisco Cánovas (Universidad de Málaga, UMA, Spain)

Leopoldo Sanchez, Catherine Bastian and Christophe Plomion (Institut National de la Recherche Agronomique, INRA, France)

Luc Harvengt (Institut Technologique Forêt Cellulose Boisconstruction Ameublement, FCBA, France)

Pär Ingvarsson (Umea University, UMU, also including Sara Von Arnold from Swedish University of Agricultural Sciences, SLU, Sweden)

Yves Van de Peer (Flanders Institute for Biotechnology, VIB, Belgium)

Berthold Heinze (Federal Research and Training Centre for Forests, Natural Hazards and Landscape, BFW, Austria)

Outi Savolainen (University of Oulu, UOULU, Finland)

Giovanni G. Vendramin (Italian National Research Council, CNR-Firenze, Italy)

Célia Miguel (Instituto de Biologia Experimental e Tecnológica, IBET, also including Jorge Paiva from Forest Center of the Tropical Research Institute, Portugal)

John Woolliams (University of Edinburgh, UEDIN, United Kingdom)

Marco Bink (Stichting Dienst Landbouwkundig Onderzoek, DLO, The Netherlands)

Carl Gunnar Fossdal (Norwegian Forest and Landscape Institute, NFLI, Norway)

David Torrents (Barcelona Supercomputing Center, BSC, Spain)

Steve Lee (Forest Research, FR, United Kingdom)

John MacKay (Université Laval, ULaval, Canada)

Kermit Ritland (University of British Columbia, UBC, Canada)

Jeffrey Dean (University of Georgia, UGA, US)

Daniel Peterson (Mississippi State University, MS State, US).

